# Experimental Social Stress: Dopaminergic Receptors, Oxidative Stress, and c-Fos Protein Are Involved in Highly Aggressive Behavior

**DOI:** 10.3389/fncel.2021.696834

**Published:** 2021-08-17

**Authors:** Renata M. Felippe, Gabriel M. Oliveira, Rafaela S. Barbosa, Betina D. Esteves, Beatriz M. S. Gonzaga, Samuel I. M. Horita, Luciana R. Garzoni, Daniela G. Beghini, Tânia C. Araújo-Jorge, Viviane M. S. Fragoso

**Affiliations:** ^1^Laboratory of Innovations in Therapies, Education and Bioproducts, Fundação Oswaldo Cruz, Rio de Janeiro, Brazil; ^2^Laboratory of Cell Biology, Fundação Oswaldo Cruz, Rio de Janeiro, Brazil; ^3^Laboratory on Thymus Research, Fundação Oswaldo Cruz, Rio de Janeiro, Brazil

**Keywords:** aggressive, stress, dopamine, reactive oxygen species, c-Fos

## Abstract

Aggression is defined as hostile behavior that results in psychological damage, injury and even death among individuals. When aggression presents itself in an exacerbated and constant way, it can be considered escalating or pathological. The association between social stress and the emergence of exacerbated aggressiveness is common and is suggested to be interconnected through very complex neurobiological factors. For example, alterations in the expression of the dopaminergic receptors D1 and D2, reactive oxygen species (ROS) and the c-Fos protein in the cortex have been observed. Our objective was to analyze which factors are involved at the neurobiological level in the highly aggressive response of Swiss Webster adult male mice in a vivarium. In this work, we investigated the relationship among dopaminergic receptors, the production of ROS and the expression of c-Fos. Mice with exacerbated aggression were identified by the model of spontaneous aggression (MSA) based on the grouping of young mice and the regrouping of the same animals in adulthood. During the regrouping, we observed different categories of behavior resulting from social stress, such as (i) highly aggressive animals, (ii) defeated animals, and (iii) harmonic groups. To evaluate the dopaminergic system and the c-Fos protein, we quantified the expression of D1 and D2 dopaminergic receptors by Western blotting and fluorescence immunohistochemistry and that of the c-Fos protein by fluorescence immunohistochemistry. The possible production of ROS was also evaluated through the dihydroethidium (DHE) assay. The results showed that aggressive and subordinate mice showed a reduction in the expression of the D1 receptor, and no significant difference in the expression of the D2 receptor was observed between the groups. In addition, aggressive mice exhibited increased production of ROS and c-Fos protein. Based on our results, we suggest that exacerbated aggression is associated with social stress, dysregulation of the dopaminergic system and exacerbated ROS production, which leads to a state of cellular oxidative stress. The overexpression of c-Fos due to social stress suggests an attempt by the cell to produce antioxidant agents to reduce the toxic cellular concentration of ROS.

## Introduction

As defined by the World Health Organization, aggression is the act(s) by which an individual harms or injures another or others of his or her own kind (Organização Mundial da Saúde [OMS], 2017). Aggression is considered pathological when it is exacerbated or constant and is observed in the behavior of individuals who have been subjected to a traumatic event and individuals who are under chronic social stress (Blair, 2003; Jakupcak et al., 2007; Tielbeek et al., 2018). Chronic social stress is a strong risk factor for the development of psychological disorders such as anxiety and posttraumatic stress disorder (McCall et al., 2015; Li et al., 2018). A symptom of these psychiatric illnesses can be impulsive aggressive behavior, which can lead the individual to acts of violence (Cassiello-Robbins et al., 2015; Yang et al., 2015; Diliberto and Kearney, 2016; Miles et al., 2016; Pinna et al., 2016).

Violence is defined as the use of force (or power) against oneself, against another person or against a group and is characterized by the possibility of resulting in physical injury, psychological damage and death (Organização Mundial da Saúde [OMS], 2017). In Brazil, there were 62,517 registered homicide cases in 2016 alone. This value is equivalent to a rate of 30.3 deaths per 100,000 inhabitants, corresponding to 30 times the European rate (Instituto de Pesquisa Aplicada [IPEA], 2018). In humans, young male individuals have a higher incidence of promoting violence (Liu et al., 2013; Murray et al., 2013).

In mice, violence is studied through the analysis of aggressive behavior (Blanchard et al., 1988). However, there is still no consensus among researchers regarding the causes of escalating, exacerbated and constant aggression in laboratory animals, mainly in mice. The difficulty in reaching a consensus is based on a complex network of factors related to genetics, biochemistry, neuroanatomy and physiology that determine the occurrence of aggressive episodes between individuals of a particular group (Batista et al., 2012; Campos et al., 2017).

The model of spontaneous aggression (MSA) is based on the grouping of mice during adolescence and regrouping in adulthood. The regrouping of mice in adulthood is a stressful social situation for animals, and they react to this stressor in a distinct and individualized manner (Batista et al., 2012; Fragoso et al., 2016; Campos et al., 2017).

Stress can cause changes in brain functioning and in the release and/or production of neurotransmitters, such as serotonin, GABA, glutamate and dopamine. Dopamine can influence the development of aggressive behavior through the mesolimbic and mesocortical pathways, as these pathways are involved in feelings of reward and pleasure (Berridge and Kringelbach, 2015). Studies show that D1R and D2R are involved in the appearance of aggressive behavior. D2 and D1 receptors are strongly expressed in the striatum region and are known to stimulate aggressive antisocial behavior, although D1R is more highly expressed in the cortex and is known to stimulate prosocial behavior (Plavén-Sigray et al., 2014, 2018).

In addition, stress can lead to mitochondrial dysfunction, resulting in an increased concentration of ROS and leading to a state of oxidative stress. These reactive oxygen species can stimulate the activation of nuclear proteins and genes that act as transcription factors, as is the case with the c-Fos protein (Phull et al., 2018; Stanisavljevic et al., 2019).

The c-Fos protein is an oncogene of the class of immediately activated genes [immediate early genes (IEGs)], belonging to a large family of FOS transcription factors. These factors have a high DNA binding affinity and are able to manipulate the transcription of specific genes (targets); through the formation of the activating protein-1 (AP1) complex, they can promote the transcription of proteins and/or other transcription factors that regulate cellular responses to stress, such as by influencing the production of antioxidant agents in the cell (Espinosa-Diez et al., 2015; Úbeda-Contreras et al., 2018). The c-Fos protein, when analyzed in animal models to verify the effect of stress on the central nervous system and the appearance of aggressive behavior, is generally elevated in the region of the medial prefrontal cortex (mPFC) (Haller et al., 2006; Biro et al., 2017; Piao et al., 2017). Some studies claim that the medial PFC region is involved in hierarchical socially dominant behavior and that dominant mice show a significant increase in the expression of c-Fos in the prelimbic subregion of the PFC (Wang et al., 2011, 2014; Goodell et al., 2017).

The objective of this study was to investigate the relationship among social stress, neurobiological disorders and exacerbated aggressive behavior in mice. We showed the importance of dopamine and impaired mitochondrial activity in the cerebral cortex of aggressive MSA mice. In this study, we further investigated the neurobiology of these animals by evaluating the expression of dopaminergic receptors, the production of ROS and the expression of the c-Fos protein in the cerebral frontal cortex of Swiss Webster mice under social stress.

## Materials and Methods

### Animals

Male Swiss Webster mice (*n* = 30/assay) were obtained from the Institute of Science and Technology in Biomodels (ICTB/Fiocruz) and were housed at the vivarium of the Laboratories of Cell Biology and Innovations in Therapies, Education and Bioproducts (LBC/LITEB—IOC). Animals adapted to the environment for 7 days under stable temperature (20–22°C), humidity (40–60%), and noise (60 dB) conditions with a 12 h light/dark cycle regulated by ventilated shelves. Food was offered *ad libitum*.

### Model of Spontaneous Aggression (MSA)

**FIGURE 1:**
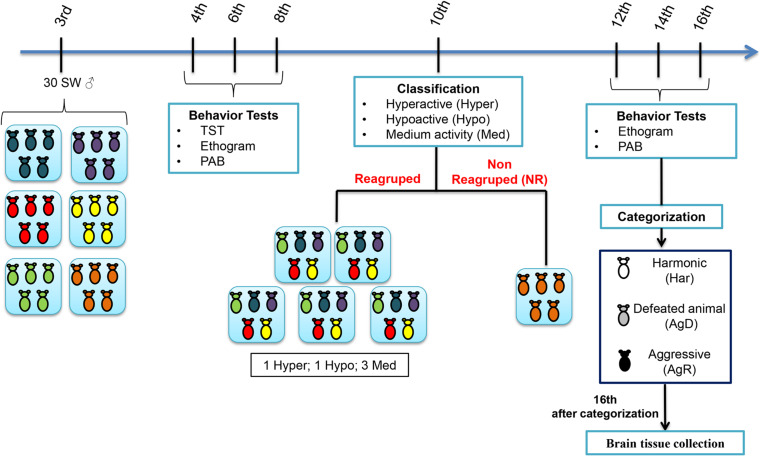
Experimental design of the model of spontaneous aggression (MSA). Grouping of the male Swiss Webster mice was performed at the 3rd week of life in five groups (*n* = 5 in each group) and behavioral tests such as the tail suspension test (TST), ethogram and pattern of aggressive behavior (PAB) test at the 4th, 6th, 8th weeks of life. At the 10th week of life, through mobility in the TST, they were classified as hyperactive (High), hypoactive (Low) and medium mobility (Med) and selected for regrouping. Some of the animals were regrouped in five groups with one High animal, one Low animal and three Med animals in each group. Regrouping represents a situation of social stress for animals. One group of 5 animals was not regrouped (NR); they were the social stress negative control. In the 12th, 14th, and 16th weeks of life, we performed the aggressive ethogram and the PAB test. In the 16th week of life, the regrouped animals were categorized as harmonic (Har), they were the social stress positive control, defeated animals (AgD) and highly aggressive (AgR). Then, the animals were euthanized, and the cerebral frontal cortices were removed for immunohistochemistry, dihydroethidium assay and Western blot analysis.

The animals at 3 weeks old (wko) were divided randomly into 6 groups (A1–A6), with 5 animals each, individually identified (C1–C5). At 4, 6, and 8 wko, we performed the tail suspension test (TST), as described by Steru et al. (1985), to map the activity profile for each animal during the grouping; an ethogram, and a pattern of aggressive behavior (PAB) test also was performed. At 10 wko, we regrouped these animals into 5 groups (R1–R5) containing 5 animals each using the TST results to standardize the regroup in each cage:1 hyperactive individual (Hyper), 1 hypoactive individual (Hypo), and 3 medium animals (Med). A cage containing 5 animals not regrouped (NR) became our negative control for social stress. At the 12th, 14th, and 16th weeks of life, the ethogram and PAB tests were repeated, and the animals were categorized as harmonic (Har), animals with reduced aggression and discrete or absent bites/lesions on the body; these animals became our positive control and considered resilient individuals; defeated animals (AgD), who presented moderate lesions on their body resulting from fights and acts of aggressions; and aggressors (AgR), animals who showed high aggressiveness in relation to other individuals in the group, facilitating the reproducibility of aggressive behavior among animals. After categorization, all animals were euthanized, and brain tissue samples were collected (Figure 1). The animal study was reviewed and approved by the Animal Use Ethics Committee of the Oswaldo Cruz Institute (CEUA/IOC) under license number CEUA/IOC-032/2019-A1.

### Tail Suspension Test (TST)

During grouping, the animals were suspended by the final third of their tail at the top of the apparatus compartment described by Steru et al. (1985), and the reaction time of the animal to this situation was quantified, in which the animal maintained a posture of mobility/immobility for 5 min per test. Three categories were statistically defined depending on the animal’s physical reaction: (a) low activity (hypoactive), 104–150 s of immobility; (b) median activity (median), between 51 and 103 s of immobility; and (c) high activity (hyperactive), 0–50 s of immobility.

### Ethogram and Pattern of Aggressive Behavior (PAB)

As described by Fragoso et al. (2016), in the grouping and regrouping phase, we carried out an aggression ethogram characterized by continuous filming (top view) using a Canon PowerShot SX20 IS^®^ camera (Lake Success, New York, United States). Each group of animals was filmed for 30 min and assessed by quantifying the number of attacks and measuring the extent of the lesion with a digital caliper (Digimess-China), making it possible to calculate the area (cm^2^) injured by the attacks. The PAB test was performed through an individual physical inspection of all animals, enabling the quantification of aggression according to predetermined scores: (0) mice with no bites or injuries on the body; (1) reduced aggressive non-sexual events and discrete bite signs on the back or tail; (2) aggressive events and moderate lesions on the tail, back and scrotum; and (3) severe events of aggression and marked lesions on the tail, back and scrotum.

### Western Blotting

The mice were euthanized by cervical dislocation, and the brain was immediately collected and immersed in cooled saline solution. Brain samples from the frontal cortex of NR, Har, AgD, and AgR animals were collected (according to Paxinos and Watson, 2007) and homogenized with lysis buffer for protein extraction (25 mM Tris HCL, 150 mM NaCl, 1 mM EDTA, 4 mM NaF, 1 mM NaVO_4_, 1% Triton X-100, and 1 mM PMSF in water, pH 7.6) plus protease and phosphatase inhibitors (Sigma-Aldrich). The samples (*n* = 10 per category) were centrifuged (5 min, 1,000 rpm, room temperature), and protein dosing was performed through spectrophotometry using a BCA kit (bicinchoninic acid) (Invitrogen Brazil). They were diluted in sample buffer composed of 80 mM Tris-HCl pH 6.8, 2% SDS, 12% glycerol, 5% β-mercaptoethanol and 0.05% bromophenol blue, subjected to 100°C for 5 min, and placed on cooling plates. The proteins were separated on a 10% polyacrylamide gel with sodium dodecyl sulfate (SDS-PAGE) containing 20 μg of protein in each well and transferred to nitrocellulose membranes.

The membranes were incubated with blocking solution (5% skimmed milk in 0.1% TBS-T) for 1 h at room temperature and subsequently with antibody made in anti-GAPDH mice (reading the internal control) (Fitzgerald, code 10R-G109A), for 30 min at room temperature with a primary antibody made in rabbit anti-D1R (1:2,000) (Abcam, ab40653), for 1 h at room temperature with a primary antibody made in rabbit anti-D2R (1:1,000) (Abcam, ab130295), and then overnight at 4°C, both diluted in blocking solution. The membranes were incubated with secondary anti-mouse and anti-rabbit antibodies conjugated to peroxidase for 1 h at room temperature. The membranes were washed three times with 0.1% TBS-T after each incubation process. Peroxidase was developed for chemiluminescence using the Super Signal West Pico kit (Invitrogen Brazil). Densitometry was performed using Image Studio Lite software (Version 5.2.5, LI-COR Biosciences, NE, United States). The relative expression of the D1 and D2 receptors was normalized to the relative GAPDH expression. Values were expressed as a variation index (VI).

### Immunohistochemistry

For analysis of the c-Fos protein, the animals were anesthetized at 1 h after the behavioral tests were finished (16 who). The mice were anesthetized with a combination of ketamine (75 mg/kg) and xylazine (10 mg/kg), followed by transcardiac perfusion with 4% paraformaldehyde (PFA). The brains were collected, fixed by overnight immersion in 4% PFA, transferred and incubated again overnight with sucrose solution gradients (10–30%) in PBS until they sank. Subsequently, 7 μm cuts were made on the brains frozen in Leica cryostat (CM 1850- Germany). The parasagittal sections were processed for immunohistochemistry and washed with PBS + 0.1% Tween (PBST). The cuts underwent an antigenic recovery process, immersed in citrate buffer (pH 6.0), and placed in Pascal (Dako, United States) at 121°C for 3 min and at 90°C for 10 s. Then, the sections were washed three times in PBS with 0.1% Triton and incubated to block endogenous IgG in blocking solution (2% skim milk, 2.5% BSA, and 8% fetal bovine serum in PBS) for 1 h at room temperature. After blocking, the sections were incubated overnight at 4°C with an antibody made in rabbit anti-cFos (1:500) (Invitrogen, OSC00040W, United States) diluted in 0.1% Triton solution in PBS.

For analysis of dopamine receptors D1 and D2, the animals were euthanized and the brains were collected and sectioned at a thickness of 7 μm, as previously described. The blocking of non-specific connections was performed with a blocking solution (10% horse serum, 4% BSA in PBS) for 1 h at room temperature. After blocking, the tissue sections were incubated overnight at 4°C with an antibody made in rabbit anti-D1R (1:400) (Abcam, ab40653) and an antibody made in rabbit anti-D2R (1:200) (Abcam, ab130295) diluted in a 1% BSA solution in PBS. To detect the primary antibodies, rabbit anti-IgG Alexa 488 (Invitrogen Brazil) was used at a concentration of 1:1,000 in PBS with 1% BSA for 1 h at RT. The slides were mounted with Prolong Diamond with DAPI (Invitrogen Brazil) and visualized by a Zeiss LSM 710 confocal microscope.

Then, the sections were washed and incubated with Alexa Fluor 488 anti-rabbit goat (1:750) antibody (Thermo Fisher Scientific, United States) diluted in blocking solution for 1 h at room temperature. The sections were washed again in PBS, stained with 0.001% Evans’s blue, and incubated with DAPI (1:5,000) for 10 min at room temperature. The slides were mounted on Prolong Gold and photographed using a Zeiss LSM 710 confocal microscope. We used NR and Har animals as negative and positive controls, respectively.

The percentage and quantification by area of positive cells for D1, D2 and c-Fos receptors were performed using ImageJ software (Version 1.50, National Institutes of Health, United States).

### DHE Assay

The animals were deeply anesthetized and euthanized. Soon after, the cortex fragment was quickly collected and frozen at Optimal Cutting Temperature (OCT) (Tissue Tek, Japan) without going through any fixation procedure. After freezing, the cortex was sectioned in a cryostat into 7 μm thick sections. To analyze the production of reactive oxygen species, the dihydroethidium (DHE) assay was used. The sections were incubated with a solution of 5 μM DHE in DMEM at 37°C for 30 min with agitation at 50 rpm. Subsequently, they were washed three times at 5-min intervals with PBS. Then, they were fixed with 4% PFA for 10 min and then washed and mounted on Prolong Gold with DAPI. The slides were photographed using a Zeiss LSM 710 confocal microscope, and the quantification of superoxide production in the samples was performed using DHE’s mean fluorescence intensity (MFI) using ImageJ software (Version 1.50, National Institutes of Health, United States).

### Statistical Analysis

Data are presented as the mean ± SD or SE values for each condition. D’Agostino-Pearson normality tests were done for all values to assess their Gaussian distribution. Comparisons between groups were performed by non-parametric Kruskal-Wallis followed by Dunn’s multiple comparisons test by using GraphPad Prism software version 8.0.1 for Windows (GraphPad Software, United States). Differences of *p* < 0.05 were considered to be significant. The analysis was performed in double blind.

## Results

### Categorization of Aggressive Animals

In this study, highly aggressive animals were selected after social stress by regrouping was carried out according to the MSA, as described in Figure 1.

**FIGURE 2:**
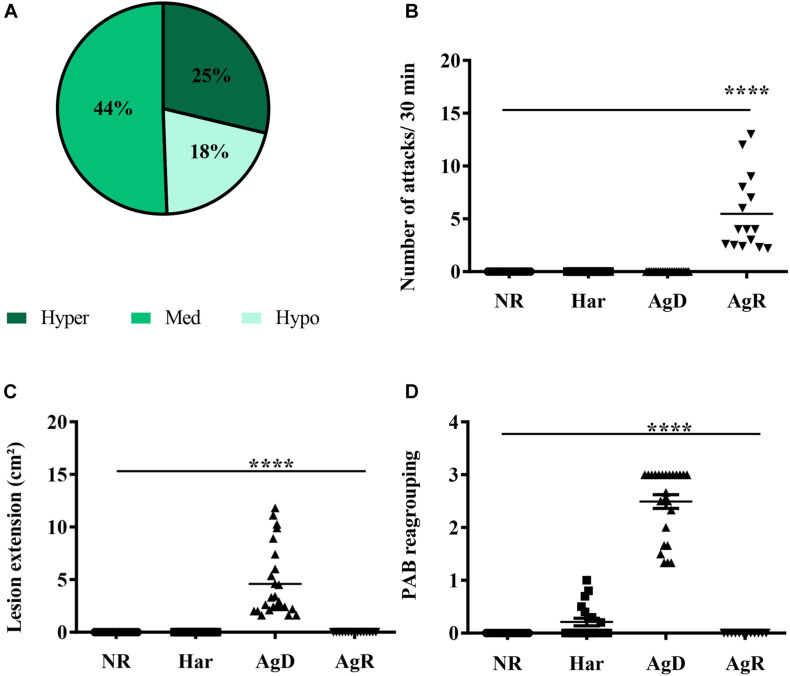
Evaluation of behavioral parameters in social stress. **(A)** Distribution of animals according to the mobility profile obtained by the TST for the regrouping in the 10th week of life. In the grouping, we obtained: Dark green: Hyperactive (25%); Green: Medium (44%); Light green: Hypoactive (18%). **(B)** Number of attacks130 min after the regrouping, according to the categorization of animals in NR (non-regrouped) (black circle), Har (harmonic) (black square), AgD (defeated) (black triangle), and AgR (aggressive) (inverted black triangle) animals. **(C)** Lesion extension (cm2) after the regrouping, according to the behavioral categories of NR, Har, AgD, and AgR animals. **(D)** Evaluation of the pattern of aggressive behavior (PAB) after the regrouping, according to the behavioral categories of NR, Har, AgD, and AgR animals. The PAB test was performed through an individual physical inspection of all animals, enabling the quantification of aggression according to predetermined scores: (0) mice with no bites or injuries on the body; (1) reduced aggressive non-sexual events and discrete bite signs on the back or tail; (2) aggressive events and moderate lesions on the tail, back, and scrotum; and (3) severe events of aggression and marked lesions on the tail, back, and scrotum. The values are expressed according to the individual results of the mice from three independent experiments (NR; *n* = 15, Har; *n* = 20, AgD; *n* = 25, AgR; *n* = 15 animals). **** corresponds to significance (*p* < 0.0001) between AgR and the other categories and AgD and the other categories by Kruskal-Wallis and Dunn’s test.

At the 4th, 6th, and 8th wko, the mobility of the grouped animals was evaluated through the TST test (Figure 2). The results showed the following: (i) 25% of the animals were hyperactive (4th wko: 22.73 ± 19.12; 6th wko: 16.36 ± 15.35, and 8th wko: 34.03 ± 30.45 s); (ii) 44% of the animals had medium mobility (4th wko: 45.37 ± 37.33; 6th wko: 78 ± 21.79, and 8th wko: 95.37 ± 25.35 s); and (iii) 18% of the animals were hypoactive (4th wko: 46.57 ± 35.52; 6th wko: 122 ± 37.42, and 8th wko: 154.93 ± 34.31 s) (Figure 2A). The hyperactivity observed in the TST results did not correlate with the development of aggressiveness. Then, the ethogram and the evaluation of the PAB were performed to analyze possible aggressive behaviors in the animals before they were exposed to the stressor factor of regrouping. The grouped animals showed low aggressiveness and no injuries; only one animal had a score of 1, and all the other animals had a score of 0. After the regrouping, the analysis of the ethogram allowed the categorization of the animals according to the level of aggressiveness: (a) Har, no aggressive behavior displayed; AgD, animals that suffered attacks; and (b) AgR, highly aggressive animals.

After introducing social stress, a significant increase in the number of attacks per 30 min was observed in AgR animals (5.5 ± 3.6 attacks/30 min) in relation to the other categories (NR: 0.0 ± 0.0; Har: 0.0 ± 0.0; AgD: 0.0 ± 0.0 attacks/30 min) (Figure 2B). Because of the number of attacks, the AgD animals presented significantly larger lesions (4.6 ± 0.6 cm^2^) than did the animals of the other categories (Figure 2C). The PAB showed that the defeated animals (AgD) had a score significantly higher than 2.0 ± 0.7, in relation to the other categories (NR: 0.0 ± 0.0; Har: 0.2 ± 0.1; AgR: 0.0 ± 0.0) (Figure 2D).

### Reduction in the Expression of the D1 Dopaminergic Receptor in the Cortex of Aggressor Animals

**FIGURE 3:**
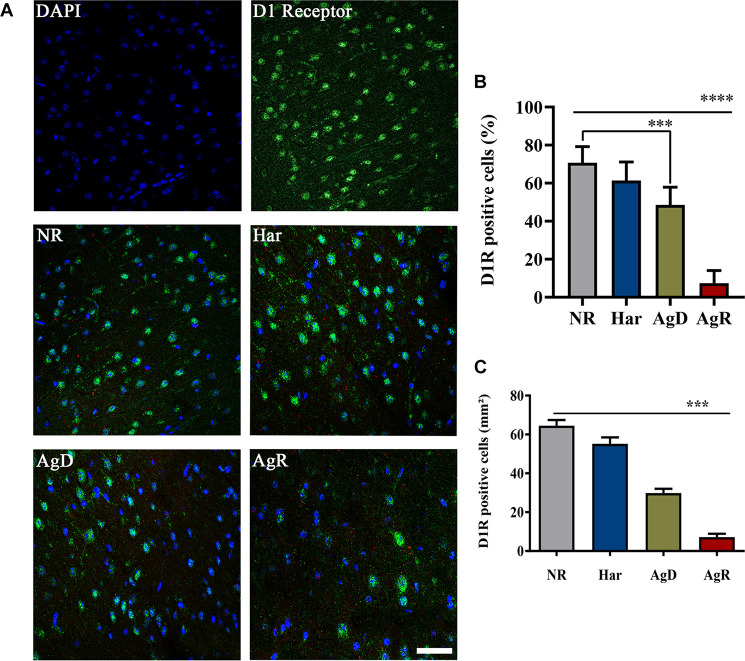
Expression of the D 1 dopaminergic receptor (D **1R)** in the cerebral frontal cortex of mice submitted to the MSA. **(A)** Representative image showing nuclear staining with DAPI (blue) and DIR staining (green). Merging of DIR and DAPI is indicative in the categories of not regrouped (NR), harmonic (Har), attacked (AgD) and aggressor (AgR) in the frontal cortex region. Bar: 50 pm. **(B)** Quantification of the percentage of cells positive for DIR. **(C)** Quantification of the extent (mm2) of the frontal cortex area marked with cells that express this receptor according to the behavioral categories: NR (gray bar), Har (blue bar), AgD (green bar) and AgR (red bar) (NR; *n* = 15, Har; *n* = 20, AgD; *n*, = 25, AgR; *n* = 15 animals). Values are expressed as the percentage and mean ± standard deviation (SD) of three independent experiments. **** corresponds to the significant difference (*p* < 0.000 1) in the percentage of positive cells between AgR and the other categories. *** corresponds to the significant difference (*p* < 0.001) of positive cells between AgR and AgD and the categories NR and Har and the difference in the percentage of positive cells between AgD and NR, by Kruskal-Wallis and Dunn’s test.

**FIGURE 4:**
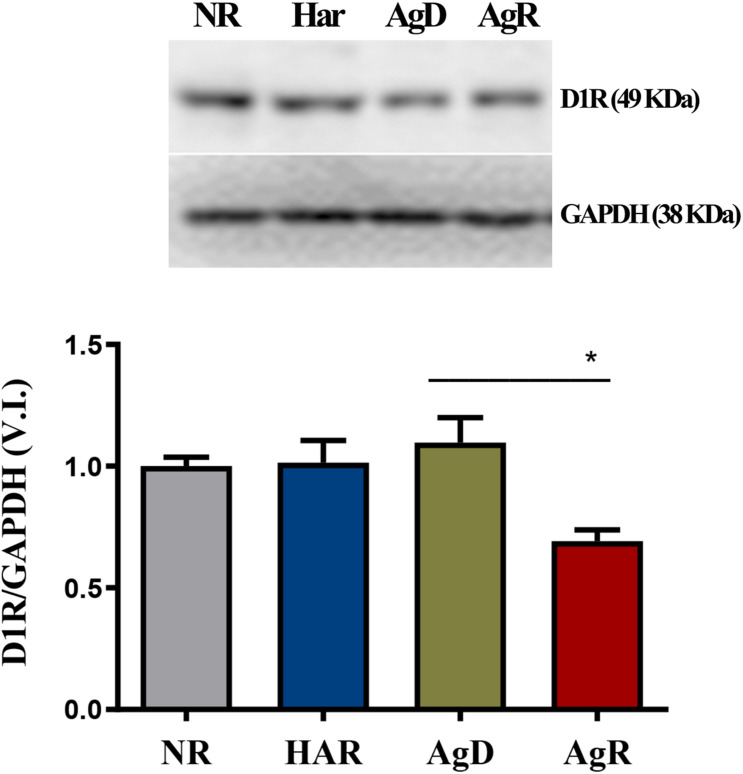
Expression levels of the Dl dopaminergic receptor. Quantitative analysis of DI receptor expression by Western blot analysis according to the categories NR (gray bar), Har (blue bar), AgD (green bar) and AgR (red bar). GAPDH was used as an internal control for the samples. The values are expressed as the variation index (V.I.) of the means * standard deviation (SD) of the analyzed samples from three independent experiments using the Kruskal-Wallis and Dunn7s test (NR; *n* = 9, Har; *n* = 10, AgD; *n* = 15; AgR; *n* = 10 animals). * corresponds to the statistical significance (*p* < 0.05) between AgR and AgD.

We investigated whether the aggressive behavior associated with social stress would be related to changes in the expression of the dopaminergic receptors D1 and D2 (Figure 3). Immunohistochemistry analysis of D1 receptor expression in the frontal cortex cerebral region showed that mice in all groups expressed this respective receptor (Figure 3A). However, only animals with highly aggressive behavior showed a significant reduction in the percentage of cells positive for the D1 receptor, approximately 7.3 ± 6.7%, compared to Har animals (61.4 ± 9.7%) and NR animals (70.7 ± 8.4%) in the cortex tissue extension (*p* ≤ 0.05). AgD animals also demonstrated a decrease in the percentage of positive cells of approximately 48% in relation to NR and Har animals at D1R (Figure 3B). Regarding the distribution of cells that express this receptor throughout the tissue of the frontal cerebral cortex, we decided to quantify in square millimeters this region of D1R-positive cells for each group. We observed that both AgD (29.86 ± 5.76 mm^2^) and AgR (7.15 ± 6.36 mm^2^) animals showed a significant reduction in D1R-positive cells compared to Har animals (55.14 ± 8.86 mm^2^) and NR animals (64.5 ± 9.25 mm^2^) (*p* ≤ 0.05). The reduction in the expression of this receptor throughout the frontal cortex was more relevant in AgR animals (Figure 3C). The assessment of D1R protein expression levels in frontal cortex cerebral tissue was performed by Western blotting to confirm this reduction (Figure 4). The results showed that the AgR mice (0.84 ± 0.39) showed a significant decrease in the expression of the D1 dopaminergic receptor when compared to the animals of the AgD group (1.009 ± 0.35). There was no significant difference between the other groups (NR: 1 ± 0.09, Har: 1.01 ± 0.27) (*p* ≤ 0.05).

**FIGURE 5:**
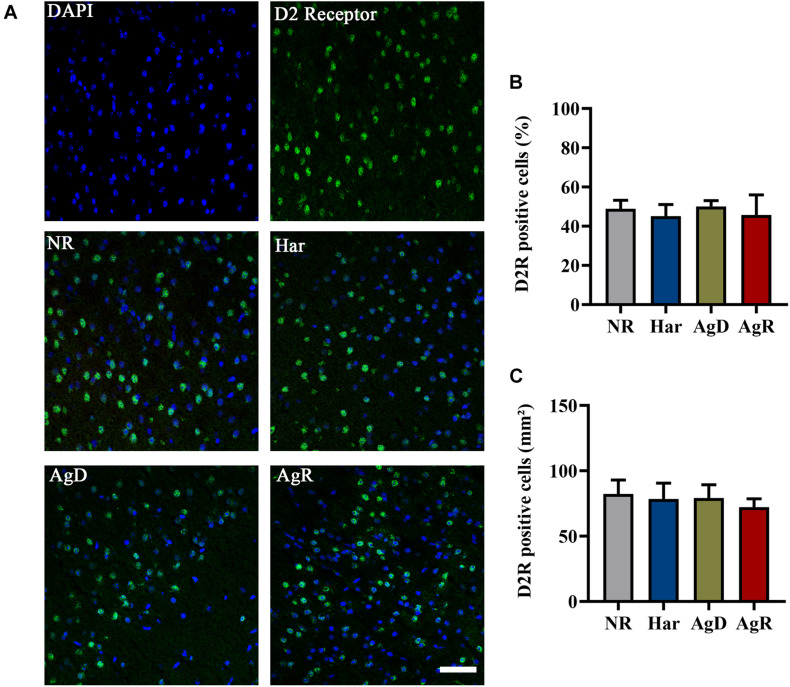
Expression of the D2 dopaminergic receptor (D2R) in the cerebral cortex of mice submitted to the MSA. **(A)** Representative image showing nuclear staining with DAPI (blue) and D2R staining (green). Merging of D2R and DAPI staining is indicative in the categories: not regrouped (NR); harmonic (Har), attacked (AgD) and aggressor (AgR) in the frontal cerebral cortex. Bars: 50 pm. **(B)** Quantification of the percentage of cells positive for D2R. **(C)** Quantification of D2R-positive cells by the extension (mm2) of the prefrontal cortex activity according to the categories NR (gray bar), Har (blue bar), AgD (green bar) and AgR (red bar) (NR; *n* = 15, Har; *n* = 20, AgD; *n* = 25, AgR; *n* = 15 animals). Values are expressed as percentage and mean ± standard deviation (SD) of three independent experiments by Kruskal-Wallis and Dunn’s test.

**FIGURE 6:**
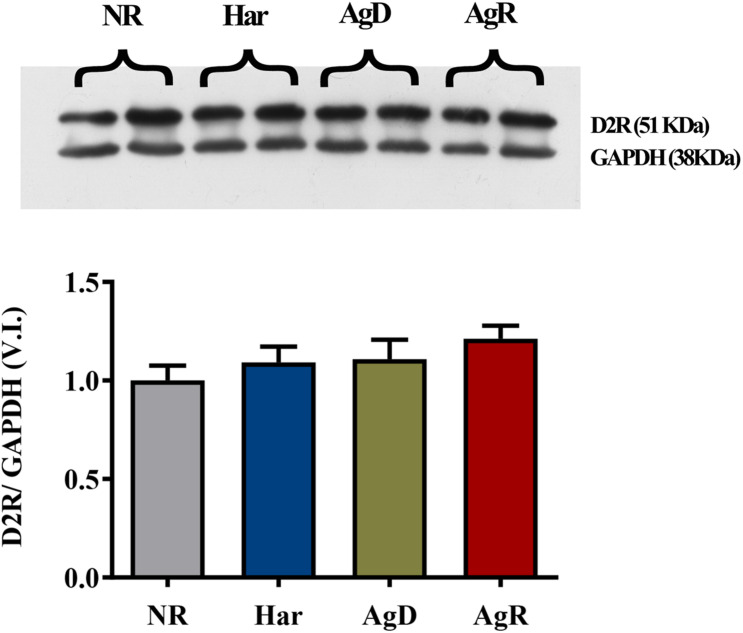
Expression levels of the dopaminergic receptor 2. Quantitative analysis of D2 receptor expression by Western blot analysis according to the categories NR (gray bar), Har (blue bar), AgD (green bar) and AgR (red bar). GAPDH was used as an internal control for the samples. The values are expressed as the variation index (VI) of the mean ± standard deviation (SD) of the analyzed samples from three independent experiments using Kruskal-Wallis and Dunn’s test (NR; *n* = 9, Har; *n* = 10, AgD; *n* = 15; AgR; *n* = 10 animals).

Analysis of dopaminergic D2R by immunohistochemistry (Figure 5) demonstrated that all groups of mice (NR, Har, AgD, and AgR) expressed this receptor in the frontal cortex cerebral tissue (Figure 5A). However, contrary to what was observed with the expression of D1R, we did not observe any variation in the percentage of positive cells expressing D2R between the categories (NR: 48.86 ± 10.6, Har: 45.16 ± 12.7, AgD: 50.05 ± 12.2, AgR: 45.71 ± 16.4%) (Figure 5B). To evaluate whether the levels of D2R protein expression between the groups corresponded to those observed in all of the frontal cortex tissue, we quantified in square millimeters this region of D2R-positive cells for each group. We observed that the number of cells positive for D2R in the frontal cortex region did not vary between the categories analyzed (NR: 82.3 ± 10.6, Har: 78.4 ± 12.1, AgD: 79.2 ± 10.2, AgR: 72.2 ± 6.4 mm^2^) (Figure 5C). We performed Western blot analysis of this receptor, and we evaluated the samples of the frontal cortex region of each category with the objective of obtaining a more detailed evaluation of the expression of D2R (Figure 6). In agreement with what was observed by immunohistochemistry, we did not find any significant difference in the expression of this receptor between the categories.

### Superoxide Production Was Higher in the Frontal Cortex of Aggressive Animals

**FIGURE 7:**
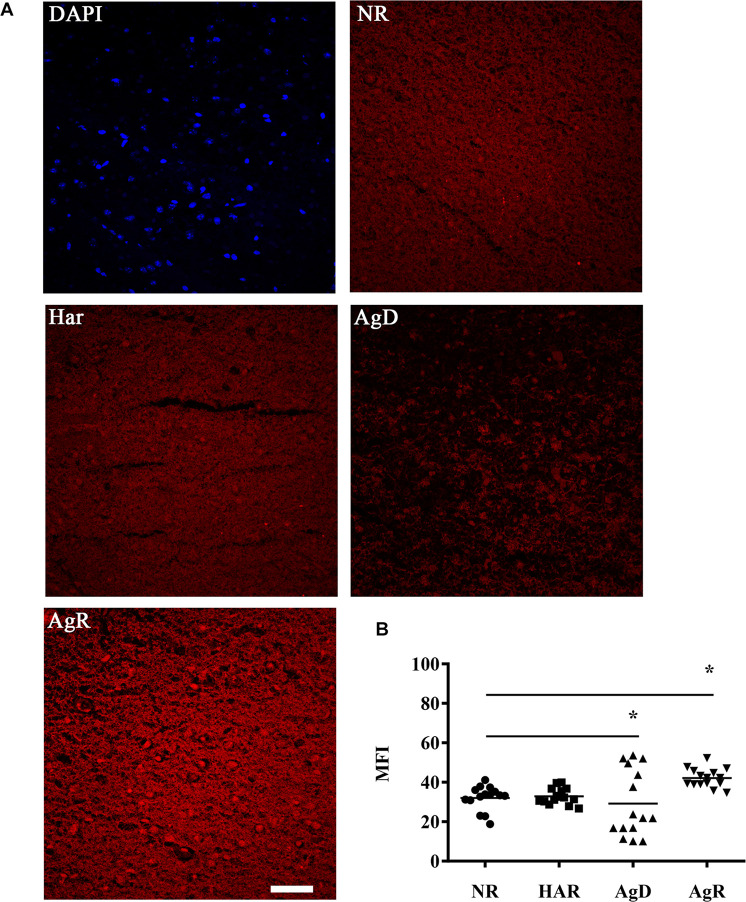
Evaluation of superoxide production in the cerebral frontal cortex of mice submitted to the MSA. **(A)** Representative image showing the production of superoxide staining (red) through the reaction of DHE with superoxide present in the tissue of the frontal cortex region in the categories not regrouped (NR), harmonic (Har), defeated (AgD) and aggressive (AgR). Bar: 50 urn. **(B)** Individual comparison of the mean fluorescence intensity (MFI) of the mice according to the categorization of animals in NR (black circle), Har (black square), AgD (black triangle) and AgR (inverted black triangle) animals in the cerebral frontal cortex (NR; *n* = 15, Har; *n* = 18, AgD; *n* = 15, AgR; *n* = 15 animals). The values are expressed according to the MFI provided by the Image J software from three independent experiments. * corresponds to statistical significance (*p* < 0.05) between AgR and the other categories and between AgD and NR and Har by Kruskal-Wallis and Dunn’s test.

Dopaminergic receptors can be compromised by mitochondrial metabolic capacity, culminating in a greater production of reactive oxygen species, mainly superoxide and hydrogen peroxides (Phull et al., 2018; Ryan et al., 2018). We investigated the changes in the production of one of the types of ROS, superoxide, in animals under social stress (Figure 7). The superoxide production analysis conducted through the DHE assay determined that all animals expressed superoxide production (Figure 7A). However, in the AgR cortex, there was a significant increase in the mean fluorescence intensity of this type of ROS from 42.1 ± 4.8 MFI when compared to the other categories (NR: 32.1 ± 6.1; Har: 32.9 ± 4.1; AgD: 29.2 ± 16.9 MFI) (*p* ≤ 0.05). The defeated mice showed a significant decrease in the production of superoxide, but this decrease was not homogeneous among animals in this category (Figure 7B). Some AgD animals that suffered more attacks during the regrouping period showed a significant increase in the production of this type of ROS (*p* ≤ 0.05), most likely as a consequence of successive attacks.

### Social Stress Increased the Expression of the c-Fos Protein

**FIGURE 8:**
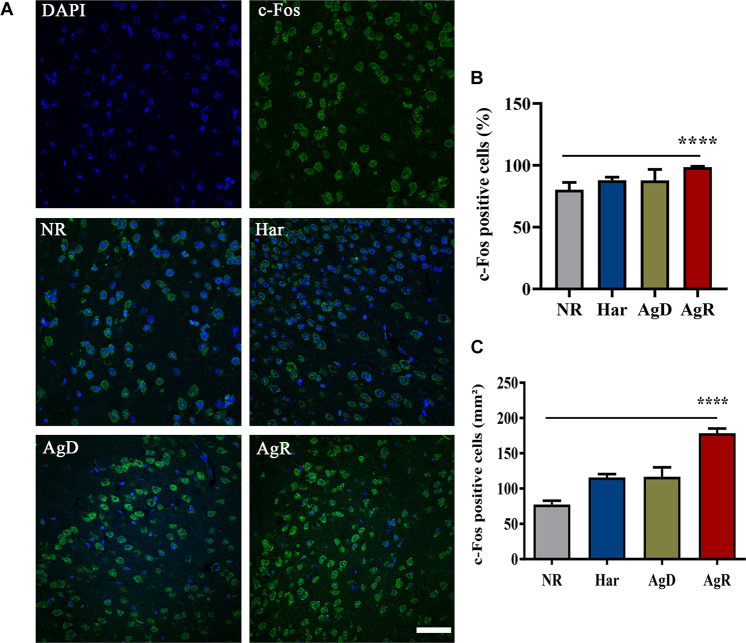
Expression of the c-Fos protein in the cerebral cortex of mice submitted to the MSA. **(A)** Representative image showing nuclear staining with DAPI (blue) and c-Fos protein staining (green). Merging of c-Fos and DAPI staining is indicative in the categories of not regrouped (NR), harmonic (Har), attacked (AgD) and aggressor (AgR) in the cerebral frontal cortex. Bar: 50 urn. **(B)** Quantification of the percentage of cells positive for c-Fos. **(C)** Quantification of c-Fos-positive cells by the extension (mm2) of the cerebral frontal cortex area according to the categories NR (gray bar), Har (blue bar), AgD (green bar) and AgR (red bar) (NR; *n* = 15, Har; *n* = 20, AgD; *n* = 25, AgR; *n* = 15 animals). Values are expressed as the percentage and mean ± standard deviation (SD) of three independent experiments. **** corresponds to the statistical significance (*p* < 0.0001) between AgR and the other categories by Kruskal-Wallis and Dunn’s test.

Some studies have described that social stress, its relationship with aggression, and increased superoxide production can also increase the expression of the c-Fos protein (Piao et al., 2017; Phull et al., 2018). In the next step, we investigated c-Fos protein expression (Figure 8). The analysis of c-Fos expression determined that all groups of mice expressed this protein (Figure 8A). However, the AgR mice showed a significant increase in the percentage of cells positive for the c-Fos protein of 98.6 ± 0.7% in relation to the other categories (NR: 80.3 ± 5.8; Har: 87.9 ± 2.5; AgD: 87.7 ± 8.9%) (*p* ≤ 0.05) (Figure 8B). Similarly, as for the c-Fos protein expression distribution, in the AgR animals, there was a significant increase in the number of cells in the cortex expressing the c-Fos protein (AgR: 178.4 ± 21.3 mm^2^) when compared to the non-regrouped animals (NR: 77.4 ± 17.0 mm^2^), harmonic (Har: 115.8 ± 14.9 mm^2^) and attacked animals (AgD: 116. 7 ± 42.2 mm^2^) (*p* ≤ 0.05) (Figure 8C).

## Discussion

In this study, we investigated whether there were changes in the expression of dopaminergic receptors (D1 and D2) and the production of superoxide and the c-Fos protein in the frontal cortex of aggressive animals experiencing social stress. Stress can be defined as the sum of external factors that affect an individual’s homeostasis (Arora et al., 2010). The organism can either adapt to the stressor or promote a maladaptive response that leads to structural changes in neural networks, which results in an imbalance in individual and social behaviors such as aggressiveness (Wooda and Bhatnagar, 2015; Wu et al., 2016; Vindas et al., 2017). Dopamine is one of the main neurotransmitters that modulates the stress response, and changes in its levels, receptors and mesolimbic and mesocortical pathways have already been identified in animals that exhibit aggressive behavior (Yu et al., 2014; Berridge and Kringelbach, 2015; Gray et al., 2015; Yamaguchi and Lin, 2018; Frau et al., 2019). In previous studies, we have shown increased levels of dopamine in the cortex, decreased concentrations of serotonin in the amygdala, striatum and hippocampus, and reduced aggressive behavior after the use of first-rate antipsychotics (haloperidol—dopaminergic D2 receptor antagonist) and second-generation antipsychotics (risperidone—dopaminergic D2 receptor antagonists and serotonergic 5-HT1A and 5-HT2A) in aggressive animals (de Oliveira et al., 2014; Fragoso et al., 2016). In these studies, we observed the importance of dopamine in the aggressive behavior of MSA individuals. Similarly, other studies have shown that in individuals under stress, aggressive impulsive responses are modulated by dopamine and serotonin. Additionally, these neurotransmitters regulate the activity of the cortex with changes in their levels and in the dopaminergic receptors in the mesolimbic and mesocortical pathways of rodents that show aggressive behavior (Nelson and Trainor, 2007; Yu et al., 2014; Berridge and Kringelbach, 2015; Gray et al., 2015; Suzuki and Lucas, 2015; Yamaguchi and Lin, 2018; Frau et al., 2019). Studies show that there is an increase in the expression of the dopaminergic D2R receptor in the cortex of animals under stress and a decrease in the expression of the D1R receptor (Plavén-Sigray et al., 2014, 2018).

The release of dopamine in the ventromedial cortex prevents the excessive activation of the hypothalamic-pituitary-adrenal (HPA) axis during stress (Tielbeek et al., 2018). In our MSA model, the levels of corticosterone did not increase in AgR mice, as was observed in the other categories (AgD and Har) of animals under social stress by regrouping (Oliveira et al., 2013). It is known that dopamine released in the cortex is capable of binding to dopaminergic receptors in the dorsal hypothalamic region, decreasing the production and release of corticotrophin releasing factor (CRF) (Di et al., 2019). CRF is involved in the activation of the HPA axis, whose activation is the main adaptive response to stress observed in animals and humans, culminating in the release of corticosterone in animals and cortisol in humans (Tielbeek et al., 2018). We believe that in the aggressor animal in our model, there may be a dysregulation of the HPA axis and an imbalance between dopaminergic neurotransmission and its receptors, preventing the development of an effective adaptive response to social stress.

Therefore, in this work, we decided to investigate dopaminergic receptors (D1 and D2). In the first analysis, we did not identify a significant difference in the expression of the D2R in the frontal cortex region among the NR, Har, AgD, and AgR categories, similar to what was observed in other studies that used the resident/intruder test as a model of aggressive behavior (Jin et al., 2015; Huang et al., 2016). However, a study carried out in young rats subjected to social isolation observed an increase in the expression of D2R in the medial prefrontal cortex, revealing that the expression of this receptor also varies according to the stressor stimulus (Han et al., 2012). In contrast, in animals subjected to passive aggressiveness, a decrease in the expression of the same receptor was observed (Suzuki et al., 2010). We suggest that in our model, the absence of a change in the expression of D2R in mice under social stress may be due to the presence of receptors in the structural conformation of dimers or isoforms. Some studies have shown that male mice considered susceptible to stress showed greater expression of dopaminergic D2R isoforms and dimers in the cortex and amygdala (Bagalkot et al., 2015; Prabhu et al., 2018).

In MSA animals under social stress by regrouping, we observed a decrease in D1R expression in AgD and AgR mice; however, this decrease was more evident in AgR animals in the frontal cortex region than in the other groups. This significant reduction in AgR is consistent with the finding of a previous study by Huang et al. (2016), who found that rodents considered susceptible to social stress showed a significant decrease in the expression of this receptor in the cortex region. In AgD animals, we believe that the decrease in D1R expression occurred because they were under intense stress due to regrouping and mainly due to the constant attacks, and the Har animals showed no changes in the expression of this receptor even under the stress of regrouping. However, we suggest that the decrease in D1R expression in the frontal cortex of the AgR group is due to an internalization process of this receptor as a result of the 85% increase in the protein concentration of dopamine in this region (de Oliveira et al., 2014). Thus, it is possible that in aggressive animals, the high levels of dopamine in the synaptic cleft have the potential for continuous binding to D1R. As an attempt to not exacerbate the cellular response, postsynaptic neurons promote desensitization followed by the internalization of D1R present in the membrane, thus decreasing the expression of these receptors in this region (downregulation) (Brunton et al., 2012). We believe that this decrease in the expression of D1 dopaminergic receptors may indirectly interfere with the decrease in the plasma concentration of corticosterone, resulting in a deficiency in the development of the adaptive response to stress in AgR animals (Oliveira et al., 2013). Another hypothesis for the reduction in the expression of this receptor on the plasma membrane could be due to the ability of D1R to interact with other receptors, such as NMDA and AMPA (Brunton et al., 2012; Offermanns and Rosenthal, 2021). Thus, D1R would be more highly expressed in the form of a heterodimer, which is not possible to be detected by the methods used in this study.

An increase in the concentration of dopamine is also able to cause a decrease in the activity of the enzyme cytochrome c oxidase (COX), compromising the mitochondrial metabolic capacity, and the dopamine released in the cortex can bind to dopaminergic receptors in the dorsal hypothalamic region, inhibiting the production and release of corticotrophin releasing factor (CRF) (Di et al., 2019). CRF is involved in the activation of the hypothalamus-pituitary-adrenal axis (HPA axis), whose activation is the main adaptive response to stress carried out by animals and humans, culminating in the release of corticosterone in animals and cortisol in humans (Czerniczyniec et al., 2007). In a recent study, our team evaluated the activity of the mitochondrial respiratory chain in the brains of animals subjected to the MSA, and we observed that in the cortex of AgR mice, the activity of the enzyme cytochrome c oxidase (COX), the important complex IV of the respiratory chain, was reduced by 43% (Mendonça et al., 2019). We know that the reduction of mitochondrial activity can promote changes in the production of reactive oxygen species (ROS) (Phull et al., 2018; Ryan et al., 2018). Therefore, in this study, we investigated whether social stress promoted changes in the production of ROS in animals subjected to the MSA. We observed a significant increase in the concentration of superoxide in AgR mice; that is, there was an exacerbated production of superoxide in the frontal cortex when compared to the other groups (AgD, Har, and NR). This suggests that the nerve cells of these animals may be in a state of oxidative stress, as has been observed in the hippocampus and prefrontal cortex in rodent neurodegenerative disease studies, corroborating our results (Shao et al., 2015; Coimbra-Costa et al., 2017; Musgrove et al., 2019). Another hypothesis for the superoxide increases in AgR animals would be that these animals may have a decrease in the production capacity of antioxidant agents (Licastro et al., 2016). Additionally, a study in rodents subjected to chronic stress showed a reduction in the total capacity of antioxidants in the prefrontal cortex (PFC) (Che et al., 2015).

Another protein that may be increased in the frontal cortex of animals under stress is c-Fos. This protein is generally used as a marker of neuronal activity. In Sprague-Dawley rats subjected to different stressors, the expression of c-Fos protein was increased in the prelimbic and infralimbic PFC subregions (Úbeda-Contreras et al., 2018). Some studies claim that the medial PFC region is involved in hierarchical socially dominant behavior and that mice considered dominant show a significant increase in the expression of c-Fos in the prelimbic subregion of the PFC (Wang et al., 2011, 2014; Goodell et al., 2017). Social stress in our experimental model also promoted a significant increase in c-Fos protein expression in the frontal cortex of AgR animals. A possible explanation for this increase may be that c-Fos protein is stimulated by an increased concentration of ROS, which in turn leads the cell to a state of oxidative stress and activates transcription factors (Phull et al., 2018). A second possibility would be that c-Fos can be stimulated through the activation of the NMDA glutamatergic receptor and Ca^2+^ channels (Stanisavljevic et al., 2019). These receptors can bind to another metabotropic receptor, such as dopaminergic D1R. In this case, we suggest that there is direct interference in the mechanism of action of this receptor since once D1R is activated, it could also activate the NMDA receptor (Thompson et al., 2016).

In our model, we believe that the aggressive responses of AgR mice are modulated by dopamine with changes in the dopaminergic receptors of the frontal cortex of these animals. We suggest that this dysregulation of the dopaminergic receptor system may inhibit the production and release of CRF and prevent the activation of the HPA axis, which could explain the absence of an increase in corticosterone with a loss in the adaptive response to stress in aggressive MSA animals. There was no significant difference in D2R expression, suggestive of receptors existing in the structural conformation of dimers or isoforms. In contrast, the D1R reduction in the frontal cortex of AgR animals could be explained by desensitization or by the formation of D1R heterodimers with NMDA and AMPA receptors, compromising their functionality and making their detection difficult by the methods used in this research.

## Conclusion

Our results suggest that an increase in dopamine associated with impaired functionality of dopaminergic receptors impairs mitochondrial metabolic capacity by decreasing COX activity. This leads the cell to a state of oxidative stress that was observed by the increase in superoxide. It is possible that the increase in reactive oxygen species (ROS) activation compromised the D1R and increased the production of c-Fos protein in the nerve cells of the cortex of AgR mice under social stress. This can explain two important points: (a) Regrouping is a marked stressor in male mice in adulthood and demonstrates individual susceptibility or resilience in stress-inducing situations. AgR, when compared to Har, showed a decreased capacity to develop an adaptive response to situations of social stress by the regrouping of mice in adulthood. (b) This failure in the ability to adapt can be observed by an exacerbated aggressiveness in behavior, with a direct relation to D1R compromise and activation of the stress response system by an increase in the production of superoxide and the expression of the c-Fos protein.

## Data Availability Statement

The original contributions presented in the study are included in the article/supplementary material, further inquiries can be directed to the corresponding author/s.

## Ethics Statement

The animal study was reviewed and approved by the Animal Use Ethics Committee of the Oswaldo Cruz Institute (CEUA/IOC) under license number CEUA/IOC-032/2019-A1.

## Author Contributions

RF, BG, and SH contributed to the methodology, formal analysis, investigation, writing—original draft, writing—review and editing, and visualization. RF, RB, BE, BG, and SH contributed to the investigation, writing—original draft, and visualization. RF, GO, BE, BG, SH, LG, DB, TA-J, and VF contributed to the writing—review and editing. RF, GO, and VF contributed to the writing—original draft. RF, GO, BG, SH, LG, DB, and VF contributed to the methodology, formal analysis, writing—review and editing, visualization, and supervision. GO, LG, DB, and VF contributed to supervision. GO, LG, TA-J, and VF contributed to the conceptualization, resources, and funding acquisition. VF contributed to the project administration. All authors read and approved the manuscript.

## Conflict of Interest

The authors declare that the research was conducted in the absence of any commercial or financial relationships that could be construed as a potential conflict of interest.

## Publisher’s Note

All claims expressed in this article are solely those of the authors and do not necessarily represent those of their affiliated organizations, or those of the publisher, the editors and the reviewers. Any product that may be evaluated in this article, or claim that may be made by its manufacturer, is not guaranteed or endorsed by the publisher.
